# Role of psychosocial status in predicting health-related quality of life at 1-year follow-up among newly diagnosed people living with HIV

**DOI:** 10.1371/journal.pone.0224322

**Published:** 2019-10-23

**Authors:** Yunxiang Huang, Dan Luo, Xi Chen, Dexing Zhang, Zhulin Huang, Shuiyuan Xiao

**Affiliations:** 1 Department of Social Medicine and Health Management, Xiangya School of Public Health, Central South University, Changsha, Hunan, People’s Republic of China; 2 Hunan Provincial Center for Disease Prevention and Control, Changsha, Hunan, People’s Republic of China; 3 The Jockey Club School of Public Health and Primary Care, Faculty of Medicine, The Chinese University of Hong Kong, Shatin, Hong Kong, People’s Republic of China; 4 Changsha Center for Disease Prevention and Control, Changsha, Hunan, People’s Republic of China; Hong Kong Polytechnic University, HONG KONG

## Abstract

**Background:**

Psychosocial problems are common among people living with HIV (PLWH) and have been cross-sectionally associated with health-related quality of life (HRQoL). We evaluated the longitudinal relationship between psychosocial status and HRQoL among PLWH during the first year after diagnosis.

**Method:**

A consecutive sample of newly diagnosed PLWH was recruited from Changsha Center for Disease Control and Prevention in Hunan Province, China. Assessments were conducted at baseline and 1 year later. The measures used in this study included the Medical Outcomes Study HIV Survey (MOS-HIV), the 9-item Patient Health Questionnaire (PHQ-9), the HIV/AIDS Stress Scale (SS-HIV) and the Social Support Rating Scale (SSRS). The trajectories of depression from baseline to 1-year follow-up were categorized into four groups: never (PHQ-9 < 10 at two time points), new-onset (PHQ-9 < 10 at baseline & PHQ-9 ≥ 10 at follow-up), recovered (PHQ-9 ≥ 10 at baseline & PHQ-9 < 10 at follow-up) and persistent depression (PHQ-9 ≥ 10 at two time points). In addition, the trajectories of stress and social support were classified by calculating the proportions of participants whose stress and social support scores changed between baseline and 1-year follow-up by more than 0.5 effect size in either direction. Generalized linear models were used to examine the potential role of baseline and changes in psychosocial status in predicting the HRQoL at 1 year, after adjusting for socio-demographic and clinical characteristics.

**Results:**

A total of 410 participants completed both the baseline and 1-year follow-up surveys. Higher stress levels at baseline predicted a lower HRQoL at 1 year, while baseline depression status and social support did not predict 1-year HRQoL scores. Compared to those who were never depressed at both baseline and follow-up, participants who experienced new-onset or persistent depression had lower HRQoL at 1 year. Additionally, the 1-year HRQoL score of participants who recovered from depression by follow-up was comparable to that of participants who were never depressed. Moreover, participants who experienced increases in stress levels by follow-up had lower HRQoL scores at 1 year than those with decreases in stress levels. Changes in social support did not predict 1-year HRQoL scores in this study.

**Conclusions:**

Assessing psychosocial status regularly and implementing effective interventions targeted at psychosocial problems may be particularly important for PLWH to improve their HRQoL. Among PLWH, special attention should be given to those with new-onset or persistent depression and those with high stress levels at the time of diagnosis and increased stress levels 1 year after the new HIV diagnosis.

## Introduction

Since the introduction of effective antiretroviral therapy (ART) in the 1990s, the morbidity and mortality of people living with HIV (PLWH) has declined dramatically [1, 2]. As HIV infection has been transformed from a rapidly fatal disease to a manageable chronic disease, the aim of treatment for PLWH is not only to prolong the survival time but also to improve health-related quality of life (HRQoL) [3]. Because of common psychosocial problems (such as the presence of depression, high stress levels and lack of social support) among PLWH, understanding the associations between psychosocial factors and HRQoL has become an increasingly important focus of studies [4, 5].

PLWH may suffer from multiple stressors, such as psychological reaction to HIV diagnosis, social stigma, concerns for disclosure, and the side effects of ART [6]. It is widely accepted that PLWH experience higher levels of stress than the general population [7]. In addition, the rate of depression is nearly twice as high in PLWH than in the seronegative population, as found in a meta-analytic review [8]. Moreover, unlike other diseases, the social support for PLWH may be limited due to the stigma of HIV infection [9]. All these situations may have a harmful impact on the HRQoL in this population [10–12].

Although the negative association between psychosocial problems and HRQoL among PLWH has been well-documented in previous studies, most studies were cross-sectional in design [13–15]. However, HRQoL and psychosocial status are dynamic phenomena that may fluctuate across different stages of the disease. The first year of receiving an HIV diagnosis is a very critical period for PLWH. A diagnosis of HIV infection itself can be a traumatic stressor for PLWH [16]. It is also a critical time period when social support is most needed. However, newly diagnosed PLWH may conceal their status for fear of possible consequences, which is a behavior that actually keeps them from accessing social support [17]. More importantly, psychosocial status among newly diagnosed PLWH may vary dramatically over the first year after diagnosis [18, 19]. For instance, in terms of individuals who were newly diagnosed, a positive change after experiencing trauma (HIV infection) may occur 1 year later in some individuals, characterized by a psychosocial adaptation to the diagnosis [20]. Some may still suffer from a negative psychological outcome resulting from a disruption in the recovery process [21]. Nevertheless, the longitudinal association between psychosocial status and HRQoL among newly diagnosed PLWH remains unclear.

Our group has previously assessed the overall changes and determinants of HRQoL among newly diagnosed PLWH [22]. We found a significant improvement of HRQoL for the overall sample 1 year after HIV diagnosis, and a significant association between HRQoL and psychosocial variables such as depression, stress and social support. This study has provided important information on the evolution of HRQoL following HIV diagnosis, and on the potential psychosocial factors that affect HRQoL. However, at least two issues remain to be addressed. First, it is unclear whether depression, stress and social support levels immediately after a new diagnosis still affect HRQoL after 1 year. Second, the role of distinct psychosocial status trajectories in the HRQoL levels at 1 year remains unknown (e.g., whether those with new-onset or persistent depression 1 year after diagnosis would have a lower 1-year HRQoL than others). Such information can be useful in preventing HRQoL from diminishing at the initial stage. Moreover, it can lead healthcare providers and policy makers to pay attention to psychosocial factors that should be intervened early in the course of HIV infection.

In light of the high level of psychosocial burden faced by individuals who were newly diagnosed with HIV and the aforementioned limitations in our previous report, the objectives of this study were (1) to examine whether psychosocial status, including depression, stress and social support at diagnosis, would predict HRQoL levels at 1 year, and (2) to determine the extent to which distinct subgroups of psychosocial status change affect HRQoL levels at 1 year, after adjusting for a range of socio-demographic and clinical characteristics.

## Methods

### Participants

This was a longitudinal study with a focus on the HRQoL of newly diagnosed PLWH. A detailed description of the study design is available elsewhere [22]. In brief, from March 1, 2013 to September 30, 2014 (baseline survey), participants were consecutively recruited from the HIV/AIDS Voluntary Counseling and Testing Clinic of the Changsha Center for Disease Control and Prevention in Hunan Province, China. Individuals who received HIV diagnosis for less than 1 month, aged 18 years or above and had lived in Changsha City for at least 6 months were eligible for this study. Information about socio-demographic, clinical and psychosocial characteristics as well as HRQoL of the participants was collected at baseline and 1 year later. The Human Research Ethics Committee of Central South University approved this study, and written informed consent was obtained from each participant before participation in this study.

There were 1267 people who received HIV diagnosis between March 1, 2013 and September 30, 2014 in Changsha. Of them, 855 met the criteria of participating in this study. A total of 557 participants were surveyed at baseline. Among them, 410 (410/557, 73.6%) completed 1-year follow-up surveys. During the 1-year follow-up period, 2 (2/147, 1.4%) participants died, 35 (35/147, 23.8%) transferred to other provincial care settings, 75 (75/147, 51.0%) refused to enter the follow-up survey, and 35 (35/147, 23.8%) could not be contacted.

### Measures

#### Outcome variables: HRQoL

HRQoL at two time points was evaluated using the Medical Outcomes Study HIV Survey (MOS-HIV), a widely used HIV-specific HRQoL instrument [23]. It includes 35 items which assess 10 dimensions of HRQoL including physical functioning (6 items), cognitive functioning (4 items), role functioning (2 items), social functioning (1 item), bodily pain (2 items), vitality (4 items), health distress (4 items), general mental health (5 items), health perception (5 items) and overall quality of life (1 item). In addition, one item assesses health transmission. The measure of each dimension is linearly transformed into a possible score ranging from 0 (worst HRQoL) to 100 (best HRQoL) on the basis of a standardized algorithm. The physical health summary scores (PHS) and mental health summary scores (MHS) could be generated from 10 dimensions according to an established scoring method. The Chinese version of MOS-HIV has good validity and reliability as demonstrated by a previous study [24].

#### Primary determinants: Depression, HIV-related stress, social support

*Depression*. The depressive symptoms experienced by participants were assessed by the 9-item Patient Health Questionnaire (PHQ-9) [25]. This instrument is a brief self-assessment questionnaire used to screen for the level of depressive symptoms over the previous two weeks, and each item is rated on a 4-point Likert scale with 0 = not at all; 1 = several days; 2 = more than half the days; and 3 = nearly every day. The total score is scored from 0 to 27, with higher scores indicating a greater severity of depressive symptoms. A cut-off score of 10 has been recommended to screen for major depressive disorder in systematic review and meta-analysis [26, 27]. The Chinese version of the PHQ-9 has good validity and reliability with a Cronbach’s *α* coefficient of 0.86 [28].

*HIV-related stress*. HIV-related stress was measured with the HIV/AIDS Stress Scale (SS-HIV), an instrument used to assess HIV-specific stress [29]. It is composed of 3 dimensions: instrumental, social and emotional stress. Each item is rated on a 5-point scale with a higher score indicating increased levels of HIV-related stress. The Chinese version of SS-HIV translated by our study groups has shown high internal consistency and test-retest reliability and adequate current validity, with an overall Cronbach’s α coefficient of 0.906 [30].

*Social support*. The Social Support Rating Scale (SSRS) was used to assess the level of social support in this study, which includes 10 items with 3 dimensions: objective support (3 items), subjective support (4 items) and use of social support (3 items) [31]. The total score is calculated by summing the scores of the 3 dimensions, with higher scores indicating greater social support. The SSRS has been widely used in China due to its high reliability and validity [32–34]. In this study, the Cronbach’s *α* coefficient for the entire scale was 0.805.

#### Covariates: Socio-demographic and clinical characteristics

Socio-demographic characteristics, including gender, age, marital status, household registration, education, employment status, individual income per month and the mode of HIV transmission, were collected in this study. The married status was recorded as single, married, divorced and widowed. Household registration was coded as rural and urban. Level of education was categorized as high school or lower and college or higher. Employment status was defined as currently employed or not. The individual income per month was dichotomized as less than 4000 Yuan ($581) or not (corresponding to the per capita monthly income in Changsha City during the period of baseline survey). Mode of HIV transmission was categorized as: heterosexual, homosexual, and others. The group of “others” included intravenous drug use (IDU), blood products, and uncertain.

Clinical characteristics, including disease-related symptoms, CD4 counts and whether they had received ART during the follow-up period were also collected. Participants were asked in the questionnaire whether they had experienced several symptoms, as follows: persistent fever, expectorate or cough, unexplained diarrhea, unintentional weight loss, active tuberculosis, oral thrush or recurrent herpes simplex. Any other perceived disease-related symptoms were recorded. The CD4 counts information and ART status were obtained from the Chinese HIV/AIDS Comprehensive Response Information Management System.

### Data analysis

All participants who completed both the baseline and follow-up surveys were included in the analysis. The Mann-Whitney tests for continuous variables or χ^2^tests for categorical variables were used to compare baseline sample characteristics between completers and dropouts from the follow-up survey. A two-tailed *alpha* value of less than 0.05 was considered statistically significant. All statistical analyses were performed in SPSS for Windows 24.0 (SPSS; Inc., Chicago, IL, USA).

#### Changes in psychosocial status and HRQoL between baseline and follow-up

The paired *t*-tests and Wilcoxon signed-rank tests were used to assess statistically significant differences in HRQoL, depression, stress and social support between baseline and 1-year follow-up.

The conventional standard effect-size (ES) calculation was adapted to identify the magnitude of any differences in HRQoL, stress and social support, with an effect size of 0.2 considered as a small change, 0.5 as a moderate change and 0.8 or greater as a large change [35]. An effect size of 0.5 is conventionally recommended as the threshold for being clinically meaningful [36]. Therefore, trajectories of changes in HRQoL, stress and social support were classified by calculating the proportions of participants whose HRQoL, stress and social support scores changed between baseline and 1-year follow-up by more than 0.5 ES in either direction.

Additionally, the trajectories of depression were categorized into four groups based on longitudinal changes in their depression status from baseline to 1-year follow-up: those without probable depression at two time points (PHQ-9 < 10 at both time points; labeled “never”), those with new probable depression at follow-up (PHQ-9 < 10 at baseline & PHQ-9 ≥ 10 at follow-up; labeled “new-onset”), those with probable depression at baseline but no depression at follow-up (PHQ-9 ≥ 10 at baseline & PHQ-9 < 10 at follow-up; labeled “recovered”) and those with probable depression at both baseline and follow-up (PHQ-9 ≥ 10 at both time points; labeled “persistent”).

#### Potential predictive role of baseline psychosocial variables for HRQoL at 1 year

We used two separate generalized linear models to determine the potential predictive role of baseline psychosocial factors for HRQoL at 1 year, with PHS and MHS at 1 year as dependent variables and the baseline psychosocial variables as independent variables. Each model was adjusted for baseline age, gender, marital status, household registration, education, employment status, individual monthly income, HIV transmission, CD4 counts and HIV-related symptoms, as well as ART status at follow-up.

#### Potential predictive role of changes in psychosocial variables for HRQoL at 1 year

Another two different generalized linear models were also used to investigate the effects of the changes in depression, stress and social support on the PHS and MHS at 1 year, controlling for baseline age, gender, marital status, household registration, education, employment status, individual monthly income, HIV transmission, CD4 counts and HIV-related symptoms as well as ART status at follow-up.

## Results

### Baseline characteristics

No significant differences in any baseline characteristics were found between participants who completed the follow-up survey and those who did not, except for employment rates. Participants who completed the follow-up survey had a lower rate of employment than those who did not complete the follow-up survey (complete vs did not complete follow-up survey: 67.3% vs 78.2%, *p* = 0.013). The final 410 participants included in the analysis were predominately younger (median 28; interquartile ranges [IQR]: 24–36), male (91.7%), single (61.5%), and had an individual monthly income less than ‘4000 Yuan’ (67.3%). Most participants (98.1%) reported becoming infected through sexual transmission. The median CD4 counts at baseline was 357 cells/mm^3^ (IQR: 254–471), and 37.3% of participants experienced at least one HIV-related symptom at baseline. A total of 218 (53.2%) had received ART during the 1-year follow-up period. The median time from ART initiation to a 1-year follow-up survey was 6 (IQR 4–9) months. Table 1 displays the sample characteristics of participants.

**Table 1:** Baseline sample characteristics of participants.

Characteristics	Final participants	Excluded participants	*p* value
	(n = 410)	(n = 147)	
**Sex**			
Male	376 (91.7%)	139 (94.6%)	0.261 ^a^
Female	34 (8.3%)	8 (5.4%)	
**Age**, median (IQR)	28 (24–36)	29 (24–38)	0.538 ^b^
**Marital status**			
Married	110 (26.8%)	87 (59.2%)	0.159 ^a^
Divorced/Widowed	48 (11.7%)	28 (19.0%)	
Single	252 (61.5%)	32 (21.8%)	
**Residence**			
Urban	210 (50.2%)	73 (47.9%)	0.746 ^a^
Rural	200 (48.8%)	74 (50.3%)	
**Education**			
Senior or lower	222 (51.1%)	80 (54.4%)	0.954 ^a^
College or higher	188 (45.9%)	67 (45.6%)	
**Employment**			
Yes	276 (67.3%)	115 (78.2%)	**0.013** ^a^
No	134 (32.7%)	32 (21.8%)	
**Monthly income**			
≤ 4000	259 (63.2%)	80 (54.4%)	0.062 ^a^
> 4000	151 (36.8%)	67 (45.6%)	
**HIV transmission**			
Heterosexual	172 (42.0%)	54 (36.7%)	0.507 ^a^
Homosexual	230 (56.1%)	92 (62.6%)	
Other	7 (1.9%)	1 (0.7%)	
**CD4 counts**, median (IQR)	357 (254–471)	350 (258–458)	0.930 ^b^
**HIV symptom**			
Yes	153 (37.3%)	46 (31.3%)	0.191 ^a^
No	257 (62.7%)	101 (68.7%)	
**PHQ-9 score (0–27)**			
No depression (<10)	249 (60.7%)	97 (66.0%)	0.260 ^a^
Probable depression (≥10)	161 (39.3%)	50 (34.0%)	
**HIV stress**, median (IQR)	26 (15–40)	25 (14–38)	0.339 ^b^
**Social support**, median (IQR)	29 (24–34)	30 (22–34)	0.852 ^b^

^a^ Chi square test

^b^ Mann-Whitney test

#### Changes in psychosocial status

The PHQ-9 score decreased for the overall sample, from a median (IQR) of 8 (3–13) at baseline to 4 (1–8) at follow-up (*p* < 0.001), with the proportion of depressed participants (PHQ-9 score ≥10) decreasing from 39.3% to 16.1%. A statistically significant decrease in the HIV-related stress median score was also observed (27 (16–40) at baseline to 15 (8–26) at follow-up, *p* < 0.001). However, the median score of social support for the overall sample declined from 29 (24–34) at baseline to 27 (22–34) at follow-up (*p* = 0.001). The calculated ES on depression, stress and social support score was 0.56, 0.63 and 0.15, respectively.

Of the 161 participants with probable depression (PHQ-9 ≥ 10) at baseline, 118 (28.8%) participants recovered from depression, but 43 (10.5%) of them remained depressed at follow-up. Of the 248 (60.7%) with no depression at baseline, 226 remained free of depression, but 23 (5.6%) had new-onset depression. In terms of stress and social support, more than half of the participants (52.2%) experienced decreases in stress that exceeded 0.5 ES (improved) and only 9.8% experienced increases in stress that exceeded 0.5 ES (worsened). Over one-third of the participants (40.0%) responded with a decline in social support that exceeded 0.5 ES (worsened). Details about the individual variations in psychosocial status change are shown in Table 2.

**Table 2:** Individual variations in psychosocial status change.

Psychosocial status	Frequency	Baseline	Follow-up	Change score
	(%)	Median (IQR)	Median (IQR)	Median (IQR)
**Depression**				
Never	226 (55.1%)	4 (2, 6)	3 (0, 5)	-1 (-3, 1)
Recovered	118 (28.8%)	15 (12, 18)	3 (0, 6)	-11 (-14, -8)
Persistent	43 (10.5%)	17 (12, 21)	13 (11, 17)	-2 (-7, 2)
New-onset	23 (5.6%)	6 (1, 8)	13 (11, 16)	9 (6, 12)
**Stress status**				
Improved	214 (52.2%)	37 (25, 48)	13 (5, 21)	-22 (-30, -13)
Unchanged	156 (38.0%)	27 (16, 40)	15 (8, 26)	-1 (-5, 2)
Worsened	40 (9.8%)	15 (10, 23)	32 (25, 45)	17 (11, 23)
**Social support**				
Improved	98 (23.9%)	26 (19, 30)	37 (30, 42)	9 (6, 15)
Unchanged	148 (36.1%)	28 (24, 33)	28 (23, 33)	0 (-2, 2)
Worsened	164 (40.0%)	32 (27, 37)	23 (17, 27)	-8 (-12, -5)

#### Changes in HRQoL

After 1 year of being diagnosed with HIV, the mean (standard deviation [SD]) PHS and MHS significantly increased from 53.5 (7.1) and 44.2 (10.1) at baseline, respectively, to 55.0 (6.5) and 49.0 (9.3) at follow-up, respectively, (PHS: *p* = 0.009: MHS: *p* < 0.001). Although the mean HRQoL score of 410 participants increased significantly, not all participants had increases in HRQoL. After 1 year, an ES of 0.15 and 0.48 for changes in PHS and MHS was shown in our participants, respectively. Among them, 135 (32.9%) participants had an increase that exceeded 0.5 ES in PHS, while 92 (23.7%) experienced a decrease that exceeded 0.5 ES. Concerning MHS, the sample proportions with greater than 0.5 ES increases and decreases were 47.3% and 13.9%, respectively. Details about the individual variations in HRQoL change are shown in Table 3.

**Table 3:** Individual variations in HRQoL change.

HRQoL	Frequency (%)	Baseline		Follow-up		Change scores	
		Mean	SD	Mean	SD	Mean	SD
**PHS**							
Improved	135 (32.9%)	48.32	7.64	57.45	4.83	9.13	5.65
Unchanged	178 (43.4%)	56.60	4.72	56.72	4.42	0.12	1.90
Worsened	97 (23.7%)	56.86	4.85	48.26	7.02	-8.60	4.87
**MHS**							
Improved	194 (47.3%)	38.61	9.31	52.50	8.03	13.89	7.65
Unchanged	159 (38.8%)	48.68	8.21	48.67	7.97	-0.01	2.64
Worsened	57 (13.9%)	50.49	7.43	38.36	8.48	-12.53	7.40

#### The predictive role of baseline psychosocial status for HRQoL at 1 year

Of the baseline data of psychosocial variables, only baseline stress was significantly associated with HRQoL at 1 year. Higher levels of stress at baseline predicted a lower PHS and MHS at 1 year. Baseline depression status and social support did not predict the HRQoL at 1 year (Table 4).

**Table 4:** The effect of baseline psychosocial status on HRQoL at 1 year ^a^.

Psychosocial status	PHS		MHS	
	β coefficient (95% CI)	*p* value	β coefficient (95% CI)	*p* value
**Baseline depression**				
No	Ref		Ref	
Yes	-0.17 (-1.67; 1.33)	0.822	-0.15 (-2.28; 1.99)	0.893
**Baseline stress**	-0.05 (-0.09; -0.01)	**0.028**	-0.13 (-0.19; -0.07)	**< 0.001**
**Baseline social support**	0.05 (-0.04; 0.13)	0.275	0.11 (-0.01; 0.22)	0.081

^a^ All models were adjusted for baseline age, gender, marital status, household registration, education, employment status, monthly income, HIV transmission, CD4 counts and symptoms, as well as ART status at follow-up.

#### The predictive role of changes in psychosocial status for HRQoL at 1 year

Compared to participants who had never had depression, participants who developed new-onset depression had lower PHS and MHS at 1 year (β = -7.13, *p* < 0.001; and β = -8.21, *p* = 0.005, respectively). Participants who reported persistent depression also showed lower PHS and MHS at 1 year (β = -7.08, *p* < 0.001; and β = -12.82, *p* < 0.001, respectively). Notably, there were no statistically significant differences in PHS and MHS at 1 year between participants who recovered from depression and those who reported no depression at the two time points. In terms of changes in stress, compared to participants who experienced decreased stress levels (improved), participants with increased stress levels (worsened) had lower PHS and MHS at 1 year (β = -3.12, *p* = 0.004; and β = -5.55, *p* < 0.001, respectively). The differences in PHS and MHS at 1 year between the different change groups of social support had no statistical significance. Details about the effect of changes in psychosocial status on HRQoL at 1 year are shown in Table 5.

**Table 5:** The effect of changes in psychosocial status on HRQoL at 1 year ^a^.

Changes in psychosocial status	PHS		MHS	
	β coefficient (95% CI)	*p* value	β coefficient (95% CI)	*p* value
**Depression**				
Never	Ref		Ref	
Recovered	-0.85 (-2.24 to 0.54)	0.231	-1.48 (-3.44 to 0.48)	0.139
New-onset	-7.12 (-9.59 to -4.64)	**< 0.001**	-8.37 (-11.86 to -4.87)	**< 0.001**
Persistent	-7.08 (-8.98 to -5.18)	**< 0.001**	-12.83 (-15.52 to -10.14)	**< 0.001**
**Stress**				
Improved	Ref		Ref	
Unchanged	-1.40 (-2.65 to -0.14)	**0.029**	-1.06 (-2.84 to 0.71)	0.238
Worsened	--3.12 (-5.13 to -1.11)	**0.002**	-5.55 (-8.38 to -2.71)	**< 0.001**
**Social support**				
Improved	Ref		Ref	
Unchanged	-0.70 (-2.16 to 0.76)	0.348	-0.16 (-2.22 to 1.90)	0.880
Worsened	-0.47 (-1.93 to 0.99)	0.530	-1.73 (-3.80 to 0.33)	0.099

^*a*^ All models were adjusted for baseline age, gender, marital status, household registration, education, employment status, monthly income, HIV transmission, CD4 counts and symptoms, as well as ART status at follow-up.

## Discussion

In this longitudinal study of people newly diagnosed with HIV, we demonstrated that, although the HRQoL improved overall within the 1 year after diagnosis, from the individual perspective, over half of participants rated their HRQoL as remaining unchanged or having worsened. It is worth noting that the 1-year level of HRQoL differed according to different trajectories of stress and depression, which indicated that HRQoL is sensitive to changes in psychosocial status. Moreover, the baseline stress levels experienced by newly diagnosed PLWH would also independently predict HRQoL at 1 year.

An important finding is that HIV-related stress at diagnosis prospectively affected HRQoL 1 year after diagnosis. The initial diagnosis may be disruptive for the majority of individuals since they have to face a future life with HIV infection, and struggle with multiple HIV-specific stressors such as stigma, disclosure, psychological adjustment and making treatment decisions [6]. The fact that individuals who perceived higher levels of stress would be more vulnerable to poor HRQoL has been well documented in previous cross-sectional studies [11, 37]. The results from this study further highlighted that stress at the time of diagnosis had a long-term negative impact on HRQoL for at least 1 year. An appropriate management of stress shortly after HIV diagnosis may be a key component to improve HRQoL among this population.

We found a significant reduction in the stress score in the sample as a whole from baseline to 1-year follow-up. However, an increased level of stress was also observed among one in ten participants (9.8%) in this study. Participants who experienced increased stress levels over 1 year were more likely to have a lower HRQoL score at 1 year. Conversely, decreases in stress levels during the follow-up period were prospectively associated with a better 1-year HRQoL. The results from our research suggested that the levels of stress among PLWH would change over time according to different stages of the disease, and distinct change patterns would exert different influences on subsequent HRQoL outcomes. A meta-analysis examining the effect of stress-management interventions on psychological outcomes among HIV-positive adults revealed that, in PLWH, stress management significantly reduced the levels of distress and improved HRQoL [38]. Future services for PLWH should include stress management and take the dynamic stress status into account instead of solely focusing on one-time assessments of stress status.

Baseline depression status did not predict the HRQoL at 1 year in this study. Participants with probable depression at the time of diagnosis did not have a lower HRQoL score 1 year after diagnosis than those with no depression. Inconsistent with this result, a longitudinal study conducted in Uganda showed that higher levels of depressive symptoms at onset of ART predicts lower improvements in HRQoL for HIV-positive patients for as long as 18 months [39]. While Kim et al. proposed that, to have an accurate understanding of the influence of depression on HRQoL, longitudinal changes in depression should be considered rather than only one-time assessments of depression status at baseline [40]. Our findings lend support to the latter viewpoints. In this study, we found that participants who had higher levels of depressive symptoms after new diagnosis do not necessarily have a lower HRQoL at the 1-year follow-up in this study, even though they were more likely to have a lower HRQoL at baseline. The effect of changes in depression on HRQoL at 1 year should be further examined.

Instead of baseline depression, the longitudinal changes in depression are more important for the 1-year follow-up HRQoL. In this study, we found that 10.5% of the participants still suffered from persistent depression and that 5.6% had new-onset depression at follow-up. These participants who experienced new-onset or persistent depression reported poorer HRQoL at 1 year compared to those who were never depressed. Of particular importance is that the HRQoL at 1 year did not significantly differ between participants who achieved remission of depression at follow-up and those who had never had depression at the two time points. In other words, even if participants who reported a remission of depression presented a significantly lower baseline HRQoL score compared to those who were never depressed, they still achieve the same level of HRQoL at 1 year over time as those reported had never been depressed at the two time points. These results underlined the opportunity to restore HRQoL by alleviating depressive symptoms among PLWH. Furthermore, the results concerning baseline depression status did not significantly predict the HRQoL in this study, while changes in depression were significantly associated with HRQoL at 1 year remind us to be cautious about using baseline depression to predict future HRQoL in clinical practice, especially in the early stages of HIV infection.

We found that the HRQoL at 1 year was not significantly dependent on social support levels at the time of diagnosis. A prospective study among men living with HIV in the US by Jia et al. revealed that lower social support at baseline was predictive of diminished changes in HRQoL over 12 months, suggesting that enhancing the initial social support of PLWH would significantly improve PHS at the end of 12 months. While the average HIV infection duration was 8.3 years in that study, social support levels remained unchanged during the 12-month follow-up period, which might partially explain why they found significant associations between baseline social support and HRQoL change [41]. However, this result may not be generalizable to patients who experience a fluctuated social support status. Additionally, a 4-year follow-up study among HAART initiative individuals also found that changes in social support rather than baseline social support are related to HRQoL [42]. Although participants reported being HIV-diagnosed for a mean of 4.3 years in that study, which is different form the sample in our study, a statistically significant change in social support during the follow-up period has been similarly reported in that study.

From multiple regression analyses, changes in social support during the follow-up period were not associated with HRQoL at 1 year. Although there were no statistically significant differences in HRQoL scores at 1 year between different groups of changes in social support, the HRQoL scores at 1 year were still larger for the improved group. The small changes in social support within 1 year among participants (ES = 0.15) possibly contributes to the disappearance of associations between changes in social support and HRQoL. Additionally, quite a few studies demonstrated that higher social support could positively influence HRQoL by decreasing the deleterious effect of psychological distress on HRQoL, such as depression [43, 44]. We should not ignore the importance of enhancing social support for PLWH.

Few previous studies focused on each HRQoL dimension among PLWH instead of two summary scores of HRQoL (i.e. PHS and MHS) [45]. While Revicki et al. mentioned that the use of summary scores rather than multiple subscale scores could simplify data analysis and the interpretation of findings from clinical trials. Moreover, these two summary scores could aid in comparisons across studies [46]. Therefore, in our study, we used the PHS and MHS scores as outcome variables. Nevertheless, there is also another possibility that baseline or change in depression, stress and social support scores would be associated with different HRQoL dimensions. This information is likely to provide another important information to improve HRQoL among PLWH. We therefore also tried to conduct multivariate analyses, with the 1-year follow-up score of each HRQoL domain designed as dependent variables, baseline or changes in psychosocial variables as independent variables. However, we found that baseline stress, changes in stress and depression were associated with almost all domains of HRQoL at 1 year, while baseline depression, as well as baseline and changes in social support were not associated with any HRQoL domains (S1 Table), which are in line with the results regarding two summary scores.

These findings indicate that the HIV-related stress and depression might influence individuals’ subjective satisfaction on each aspect of the HRQoL, highlighting the importance of being aware of the depression and stress faced by PLWH. The result that social support was not significantly associated with any dimension of HRQoL in this study suggests that the influence of social support on HRQoL during the early post-diagnosis phase may be relatively smaller compared to the influence of depression and stress levels, which is not surprising considering the traumatic experience of being newly diagnosed with HIV. A prospective study design with a longer observation period may be helpful to give a deeper understanding of the association between social support and HRQoL.

Several limitations in the study should be considered. First, our sample was based on a convenience sampling method, which may limit the generalizability of our findings. Second, it should be noted that a proportion of participants did not complete a 1-year follow-up survey. These participants may display a different psychosocial status and HRQoL at follow-up, which might bias the results. Another limitation is the possibility of a tautological relationship between psychosocial variables (such as stress and depression) and the psychological outcome measures of the MOS-HIV. The strong association between those two sets of variables could be partially attributed to conceptual overlap. Additionally, only two time points were investigated in this study, which could not reflect the changes in the psychosocial status of PLWH in the whole HIV disease trajectory, and the effect of psychosocial factors on HRQoL may be different at various stages of the infection. Long-term assessment of psychosocial status and HRQoL among PLWH should be performed in future studies.

## Conclusion

As HIV infection becomes a chronic illness, studies need to focus on the dynamic trend of psychosocial status and HRQoL rather than only one-time assessment. Stress levels at the time of diagnosis, along with changes in stress and depression status within 1 year, could prospectively influence the HRQoL 1 year after diagnosis among newly diagnosed PLWH. Assessing psychosocial status regularly and implementing effective interventions targeted at psychosocial problems may be particularly important for this population to improve HRQoL. Among PLWH, special attention should be paid to those with new-onset or persistent depression and those with high baseline stress levels and increased stress levels 1 year after the new HIV diagnosis.

## Supporting information

S1 Dataset Data used in this analysis. (XLSX)

S1 Table The effect of baseline and changes in psychosocial status on each of the 10 HRQoL domains at 1 year. (DOCX)

## References

[1] Michaels SH, Clark R, Kissinger P. Declining morbidity and mortality among patients with advanced human immunodeficiency virus infection. The New England journal of medicine. 1998;339(6):405–6. 10.1056/NEJM199808063390612 MEDLINE:9696654. 9696654

[2] Samji H, Cescon A, Hogg RS, Modur SP, Althoff KN, Buchacz K, et al Closing the Gap: Increases in Life Expectancy among Treated HIV-Positive Individuals in the United States and Canada. PLoS One. 2013;8(12). 10.1371/journal.pone.0081355 WOS:000328740300004. 24367482 PMC3867319

[3] Carpenter CCJ, Cooper DA, Fischl MA, Gatell JM, Gazzard BG, Hammer SM, et al Antiretroviral therapy in adults—Updated recommendations of the International AIDS Society-USA Panel. Jama-Journal of the American Medical Association. 2000;283(3):381–90. 10.1001/jama.283.3.381 WOS:000084732400031. 10647802

[4] Pozniak A. Quality of life in chronic HIV infection. Lancet HIV. 2014;1(1):e6–7. Epub 2015/10/02. 10.1016/S2352-3018(14)70003-7 . 26423817

[5] Heywood W, Lyons A. HIV and Elevated Mental Health Problems: Diagnostic, Treatment, and Risk Patterns for Symptoms of Depression, Anxiety, and Stress in a National Community-Based Cohort of Gay Men Living with HIV. AIDS Behav. 2016;20(8):1632–45. Epub 2016/02/15. 10.1007/s10461-016-1324-y . 26874848

[6] Martinez J, Lemos D, Hosek S, Adolescent Med Trials N. Stressors and Sources of Support: The Perceptions and Experiences of Newly Diagnosed Latino Youth Living with HIV. AIDS Patient Care STDS. 2012;26(5):281–90. 10.1089/apc.2011.0317 WOS:000303435800006. 22536931 PMC3335135

[7] Garrido-Hernansaiz H, Alonso-Tapia J. Associations Among Resilience, Posttraumatic Growth, Anxiety, and Depression and Their Prediction From Stress in Newly Diagnosed People Living With HIV. Janac-Journal of the Association of Nurses in Aids Care. 2017;28(2):289–94. 10.1016/j.jana.2016.12.005 WOS:000396440700010. 28109699

[8] Ciesla JA, Roberts JE. Meta-analysis of the relationship between HIV infection and risk for depressive disorders. Am J Psychiatry. 2001;158(5):725–30. 10.1176/appi.ajp.158.5.725 WOS:000168479000011. 11329393

[9] Pichon LC, Rossi KR, Ogg SA, Krull LJ, Griffin DY. Social Support, Stigma and Disclosure: Examining the Relationship with HIV Medication Adherence among Ryan White Program Clients in the Mid-South USA. Int J Environ Res Public Health. 2015;12(6):7073–84. 10.3390/ijerph120607073 WOS:000357268500084. 26103592 PMC4483749

[10] Friedland J, Renwick R, Mccoll M. Coping and social support as determinants of quality of life in HIV/AIDS. AIDS Care. 1996;8(1):15–31. 10.1080/09540129650125966 MEDLINE:8664366. 8664366

[11] Au A, Chan I, Li P, Chung R, Po LM, Yu P. Stress and health-related quality of life among HIV-infected persons in Hong Kong. AIDS Behav. 2004;8(2):119–29. 10.1023/B:AIBE.0000030243.50415.c0 WOS:000221882600002. 15187474

[12] Reis RK, Haas VJ, Dos Santos CB, Teles SA, Gimenez Galvao MT, Gir E. Symptoms of depression and quality of life of people living with HIV/AIDS. Rev Lat Am Enfermagem. 2011;19(4):874–81. WOS:000293983500004. 10.1590/s0104-11692011000400004 21876938

[13] Degroote S, Vogelaers DP, Vermeir P, Mariman A, De Rick A, Van Der Gucht B, et al Socio-economic, behavioural, (neuro)psychological and clinical determinants of HRQoL in people living with HIV in Belgium: a pilot study. J Int AIDS Soc. 2013;16 10.7448/ias.16.1.18643 WOS:000328696300001. 24331754 PMC3862978

[14] Briongos Figuero LS, Bachiller Luque P, Palacios Martin T, Gonzalez Sagrado M, Eiros Bouza JM. Assessment of factors influencing health-related quality of life in HIV-infected patients. HIV Med. 2011;12(1):22–30. 10.1111/j.1468-1293.2010.00844.x WOS:000284963200004. 20497251

[15] Perez IR, Rodriguez-Bano J, Ruz MaL, Jimenez AD, Prados MC, Liano JP, et al Health-related quality of life of patients with HIV: Impact of sociodemographic, clinical and psychosocial factors. Qual Life Res. 2005;14(5):1301–10. 10.1007/s11136-004-4715-x WOS:000229642000010. 16047505

[16] Neigh GN, Rhodes ST, Valdez A, Jovanovic T. PTSD co-morbid with HIV: Separate but equal, or two parts of a whole? Neurobiol Dis. 2016;92(Pt B):116–23. Epub 2015/11/26. 10.1016/j.nbd.2015.11.012 26592355 PMC5673262

[17] Feigin R, Sapir Y, Patinkin N, Turner D. Breaking Through the Silence: The Experience of Living With HIV-Positive Serostatus, and Its Implications on Disclosure. Soc Work Health Care. 2013;52(9):826–45. 10.1080/00981389.2013.827143 WOS:000325788900003. 24117031

[18] Tao J, Vermund SH, Lu H, Ruan Y, Shepherd BE, Kipp AM, et al Impact of Depression and Anxiety on Initiation of Antiretroviral Therapy Among Men Who Have Sex with Men with Newly Diagnosed HIV Infections in China. AIDS Patient Care STDS. 2017;31(2):96–104. 10.1089/apc.2016.0214 WOS:000394343800006. 28170305 PMC5312604

[19] Garrido-Hernansaiz H, Alonso-Tapia J. Social Support in Newly Diagnosed People living With HIV: Expectations and Satisfaction Along Time, Predictors, and Mental Health Correlates. Janac-Journal of the Association of Nurses in Aids Care. 2017;28(6):849–61. 10.1016/j.jana.2017.06.007 WOS:000414218900003. 28705757

[20] Rzeszutek M. A longitudinal analysis of posttraumatic growth and affective well-being among people living with HIV: The moderating role of received and provided social support. PLoS One. 2018;13(8):e0201641 Epub 2018/08/07. 10.1371/journal.pone.0201641 30080882 PMC6078301

[21] Nightingale VR, Sher TG, Hansen NB. The impact of receiving an HIV diagnosis and cognitive processing on psychological distress and posttraumatic growth. J Trauma Stress. 2010;23(4):452–60. Epub 2010/07/22. 10.1002/jts.20554 20648562 PMC3629914

[22] Huang YX, Luo D, Chen X, Zhang DX, Wang M, Qiu YY, et al Changes and determinants of health-related quality of life among people newly diagnosed with HIV in China: a 1-year follow-up study. Qual Life Res. 2019;28(1):35–46. 10.1007/s11136-018-1998-x WOS:000456282400003. 30206817 PMC6339666

[23] Wu AW, Revicki DA, Jacobson D, Malitz FE. Evidence for reliability, validity and usefulness of the Medical Outcomes Study HIV Health Survey (MOS-HIV). Qual Life Res. 1997;6(6):481–93. Epub 1997/08/01. . 9330549 10.1023/a:1018451930750

[24] Huang Z-J, Tian M, Dai S-Y, Ye D-Q. Feasibility, reliability and validity of the Chinese simplified version of the MOS-HIV health survey among AIDS patients in China. Qual Life Res. 2013;22(2):403–7. 10.1007/s11136-012-0148-0 WOS:000315279500018. 22392524

[25] Kroenke K, Spitzer RL, Williams JBW. The PHQ-9—Validity of a brief depression severity measure. J Gen Intern Med. 2001;16(9):606–13. 10.1046/j.1525-1497.2001.016009606.x WOS:000171184700005. 11556941 PMC1495268

[26] Kroenke K, Spitzer RL, Williams JBW, Loewe B. The Patient Health Questionnaire Somatic, Anxiety, and Depressive Symptom Scales: a systematic review. Gen Hosp Psychiatry. 2010;32(4):345–59. 10.1016/j.genhosppsych.2010.03.006 WOS:000280389800001. 20633738

[27] Manea L, Gilbody S, Mcmillan D. A diagnostic meta-analysis of the Patient Health Questionnaire-9 (PHQ-9) algorithm scoring method as a screen for depression. Gen Hosp Psychiatry. 2015;37(1):67–75. 10.1016/j.genhosppsych.2014.09.009 WOS:000348042700015. 25439733

[28] Wang W, Bian Q, Zhao Y, Li X, Wang W, Du J, et al Reliability and validity of the Chinese version of the Patient Health Questionnaire (PHQ-9) in the general population. Gen Hosp Psychiatry. 2014;36(5):539–44. Epub 2014/07/16. 10.1016/j.genhosppsych.2014.05.021 . 25023953

[29] Pakenham KI, Rinaldis M. Development of the HIV/AIDS stress scale. Psychol Health. 2002;17(2):203–19. 10.1080/08870440290013680 WOS:000176007300005.

[30] Niu L, Qiu Y, Luo D, Chen X, Wang M, Pakenham KI, et al Cross-Culture Validation of the HIV/AIDS Stress Scale: The Development of a Revised Chinese Version. PLoS One. 2016;11(4). 10.1371/journal.pone.0152990 WOS:000373592100059. 27043134 PMC4820124

[31] Xiao S. The theory basis and application of the Social Support Rating Scale. Journal of Clinical Psychiatry Medicine. 1997;4(2):98–100.

[32] Cai W-P, Pan Y, Zhang S-M, Wei C, Dong W, Deng G-H. Relationship between cognitive emotion regulation, social support, resilience and acute stress responses in Chinese soldiers: Exploring multiple mediation model. Psychiatry Res. 2017;256:71–8. 10.1016/j.psychres.2017.06.018 WOS:000412787700011. 28624675

[33] Du J, Shao S, Jin G-H, Qian C-G, Xu W, Lu X-Q. Factors associated with health-related quality of life among family caregivers of disabled older adults: a cross-sectional study from Beijing. Medicine. 2017;96(44). 10.1097/md.0000000000008489 WOS:000415106800059. 29095308 PMC5682827

[34] Wu H, Wu S, Wu H, Xia Q, Li N. Living Arrangements and Health-Related Quality of Life in Chinese Adolescents Who Migrate from Rural to Urban Schools: Mediating Effect of Social Support. Int J Environ Res Public Health. 2017;14(10). 10.3390/ijerph14101249 WOS:000414763200158. 29048382 PMC5664750

[35] Cohen J, Cohen Ja, Cohen L, Cohen Jj, Cohen Jw, Cohen Ds, et al Statistical Power Analysis for the Behavioral Science. New York: Academic Press; 1977.

[36] Kazis LE, Anderson JJ, Meenan RF. Effect sizes for interpreting changes in health status. Med Care. 1989;27(3 Suppl):S178–89. 10.1097/00005650-198903001-00015 MEDLINE:2646488. 2646488

[37] Pokhrel KN, Sharma VD, Shibanuma A, Pokhrel KG, Mlunde LB, Jimba M. Predicting health-related quality of life in people living with HIV in Nepal: mental health disorders and substance use determinants. Aids Care-Psychological and Socio-Medical Aspects of Aids/Hiv. 2017;29(9):1137–43. 10.1080/09540121.2017.1332331 WOS:000405565900010. 28547996

[38] Scott-Sheldon LaJ, Kalichman SC, Carey MP, Fielder RL. Stress management interventions for HIV+ adults: A meta-analysis of Randomized controlled trials, 1989 to 2006. Health Psychol. 2008;27(2):129–39. 10.1037/0278-6133.27.2.129 WOS:000254659800001. 18377131 PMC2409585

[39] Ezeamama AE, Woolfork MN, Guwatudde D, Bagenda D, Manabe YC, Fawzi WW, et al Depressive and Anxiety Symptoms Predict Sustained Quality of Life Deficits in HIV-Positive Ugandan Adults Despite Antiretroviral Therapy A Prospective Cohort Study. Medicine. 2016;95(9). 10.1097/md.0000000000002525 WOS:000375208900025. 26945347 PMC4782831

[40] Kim S-Y, Kim S-W, Shin I-S, Park M-H, Yoon J-H, Yoon J-S, et al Changes in depression status during the year after breast cancer surgery and impact on quality of life and functioning. Gen Hosp Psychiatry. 2018;50:33–7. 10.1016/j.genhosppsych.2017.09.009 WOS:000424343000005. 28987920

[41] Jia HG, Uphold CR, Wu S, Chen GJ, Duncan PW. Predictors of changes in health-related quality of life among men with HIV infection in the HAART era. AIDS Patient Care STDS. 2005;19(6):395–405. 10.1089/apc.2005.19.395 WOS:000230459000006. 15989435

[42] Burgoyne R, Renwick R. Social support and quality of life over time among adults living with HIV in the HAART era. Soc Sci Med. 2004;58(7):1353–66. 10.1016/S0277-9536(03)00314-9 WOS:000220271300011. 14759681

[43] Bekele T, Rourke SB, Tucker R, Greene S, Sobota M, Koornstra J, et al Direct and indirect effects of perceived social support on health-related quality of life in persons living with HIV/AIDS. Aids Care-Psychological and Socio-Medical Aspects of Aids/Hiv. 2013;25(3):337–46. 10.1080/09540121.2012.701716 WOS:000314964600010. 22774876

[44] Shrestha R, Copenhaver M, Bazazi AR, Huedo-Medina TB, Krishnan A, Altice FL. A Moderated Mediation Model of HIV-Related Stigma, Depression, and Social Support on Health-Related Quality of Life among Incarcerated Malaysian Men with HIV and Opioid Dependence. AIDS Behav. 2017;21(4):1059–69. Epub 2017/01/22. 10.1007/s10461-017-1693-x 28108877 PMC5344717

[45] Jia H, Uphold CR, Zheng Y, Wu S, Chen GJ, Findley K, et al A further investigation of health-related quality of life over time among men with HIV infection in the HAART era. Qual Life Res. 2007;16(6):961–8. Epub 2007/05/01. 10.1007/s11136-007-9214-4 . 17468942

[46] Revicki DA, Sorensen S, Wu AW. Reliability and validity of physical and mental health summary scores from the Medical Outcomes Study HIV Health Survey. Med Care. 1998;36(2):126–37. Epub 1998/02/25. 10.1097/00005650-199802000-00003 . 9475468

