# Supplementary material for: Role of psychosocial status in predicting health-related quality of life at 1-year follow-up among newly diagnosed people living with HIV
Source: PLoS One. 2019 Oct 23;14(10):e0224322. doi: 10.1371/journal.pone.0224322 (PMC6808448; doi:10.1371/journal.pone.0224322)
Supplement: S1 Table — (DOCX) [file pone.0224322.s002.docx]

**Supplementary Table A: The effect of baseline psychosocial status on each of the 10 HRQoL domains at 1 year ^a^.**

| **Baseline psychosocial status** | **HRQoL domain** | | | | | | | | | |
| --- | --- | --- | --- | --- | --- | --- | --- | --- | --- | --- |
|  | General health perception | Physical functioning | Role functioning | Social functioning | Cognitive functioning | Pain | Mental health | Validity | Health distress | Quality of life |
| **Depression** |  |  |  |  |  |  |  |  |  |  |
| No | Ref | Ref | Ref | Ref | Ref | Ref | Ref | Ref | Ref | Ref |
| Yes | -0.29 | 1.10 | 2.70 | 0.25 | -0.67 | -0.45 | 1.73 | 0.15 | 0.79 | -1.54 |
| **Stress** | -0.15^*^ | -0.04 | -0.20^*^ | -0.16 | -0.17^*^ | -0.13^*^ | -0.25^*^ | -0.18^*^ | -0.26^**^ | -0.19^*^ |
| **Social support** | 0.163 | 0.16 | 0.06 | 0.003 | 0.22 | 0.07 | 0.12 | 0.25 | 0.30^*^ | 0.05 |

*^a^* All models were adjusted for baseline age, gender, marital status, household registration, education, employment status, monthly income, HIV transmission, CD4 counts and symptoms, as well as ART status at follow-up.

**Supplementary Table B: The effect of changes in psychosocial status on each of the 10 HRQoL domains at 1 year ^a^.**

| **Changes in psychosocial status** | **HRQoL domain** | | | | | | | | | |
| --- | --- | --- | --- | --- | --- | --- | --- | --- | --- | --- |
|  | General health perception | Physical functioning | Role functioning | Social functioning | Cognitive functioning | Pain | Mental health | Validity | Health distress | Quality of life |
| **Depression** |  |  |  |  |  |  |  |  |  |  |
| Never | Ref | Ref | Ref | Ref | Ref | Ref | Ref | Ref | Ref | Ref |
| Recovered | -1.27 | -2.43 | -3.47 | -2.04 | 0.02 | -2.03 | -4.85^*^ | -2.36 | -3.22 | -0.55 |
| New-onset | -11.5^**^ | -10.89^***^ | -16.20^**^ | -21.18^***^ | -10.49^**^ | -16.51^**^ | -16.73^***^ | -18.89^***^ | -16.25^***^ | -7.60^*^ |
| Persistent | -17.77^***^ | -9.44^***^ | -30.20^***^ | -17.73^***^ | -21.85^***^ | -13.43^**^ | -21.99^***^ | -20.26^***^ | -29.13^***^ | -13.16^***^ |
| **Stress** |  |  |  |  |  |  |  |  |  |  |
| Improved | Ref | Ref | Ref | Ref | Ref | Ref | Ref | Ref | Ref | Ref |
| Unchanged | -3.55 | -2.30 | -6.97^*^ | 0.08 | -2.35 | -3.86^*^ | -1.11 | -1.60 | -3.97 | -1.46 |
| Worsened | -5.95 | -5.67^*^ | -9.98^*^ | -11.38^**^ | -12.11^***^ | -5.62^*^ | -9.55^**^ | -5.74 | -13.06^***^ | -5.42^*^ |
| **Social support** |  |  |  |  |  |  |  |  |  |  |
| Improved | Ref | Ref | Ref | Ref | Ref | Ref | Ref | Ref | Ref | Ref |
| Unchanged | 1.03 | -2.01 | -3.61 | -2.50 | -0.07 | -0.88 | -2.64 | -1.92 | -0.33 | 2.47 |
| Worsened | -1.15 | -1.50 | 0.02 | -4.80 | -2.41 | -0.19 | -3.91 | -3.35 | -2.48 | -1.67 |

*^a^* All models were adjusted for baseline age, gender, marital status, household registration, education, employment status, monthly income, HIV transmission, CD4 counts and symptoms, as well as ART status at follow-up.
